# How well do you know your growth chambers? Testing for chamber effect using plant traits

**DOI:** 10.1186/s13007-015-0088-0

**Published:** 2015-09-22

**Authors:** Amanda S. Porter, Christiana Evans-Fitz.Gerald, Jennifer C. McElwain, Charilaos Yiotis, Caroline Elliott-Kingston

**Affiliations:** School of Biology and Environmental Science, Earth Institute, O’Brien Centre for Science, University College Dublin, Belfield, Dublin 4, Ireland

**Keywords:** Plant growth chamber, Controlled environment, Chamber effect, Gas analysis, Stable carbon isotopes, Chlorophyll fluorescence, Fresh weight, Plant anatomy, Experimental design, Uniformity trials

## Abstract

**Background:**

Plant growth chambers provide a controlled environment to analyse the effects of environmental parameters (light, temperature, atmospheric gas composition etc.) on plant function. However, it has been shown that a ‘chamber effect’ may exist whereby results observed are not due to an experimental treatment but to inconspicuous differences in supposedly identical chambers. In this study, *Vicia faba* L. ‘Aquadulce Claudia’ (broad bean) plants were grown in eight walk-in chambers to establish if a chamber effect existed, and if so, what plant traits are best for detecting such an effect. A range of techniques were used to measure differences between chamber plants, including chlorophyll fluorescence measurements, gas exchange analysis, biomass, reproductive yield, anatomical traits and leaf stable carbon isotopes.

**Results and discussion:**

Four of the eight chambers exhibited a chamber effect. In particular, we identified two types of chamber effect which we term ‘*resolvable’* or ‘*unresolved’*; a *resolvable* chamber effect is caused by malfunctioning components of a chamber and an *unresolved* chamber effect is caused by unknown factors that can only be mitigated by appropriate experimental design and sufficient replication. Not all measured plant traits were able to detect a chamber effect and no single trait was capable of detecting all chamber effects. Fresh weight and flower count detected a chamber effect in three chambers, stable carbon isotopes (δ^13^C) and net rate CO_2_ assimilation (A_n_) identified a chamber effect in two chambers, stomatal conductance (g_s_) and total performance index detected an effect only in one chamber.

**Conclusion:**

(1) Chamber effects can be adequately detected by fresh weight measurements and flower counts on *Vicia faba* plants. These methods were the most effective in terms of detection and most efficient in terms of time. (2) δ^13^C, g_s_ and A_n_ measurements help distinguish between *resolvable* and *unresolved* chamber effects. (3) *Unresolved* chamber effects require experimental unit replication while *resolvable* chamber effects require investigation, repair and retesting in advance of initiating further experiments.

## Background

Controlled environment plant growth chambers are invaluable in allowing researchers to determine the effects of specific biotic or abiotic parameters on plants. A wide range of plants can be grown in artificial environments where all abiotic factors can be controlled; by varying one or more of these (e.g. temperature) the effect on plants can be tested (e.g. [1–5]). Field experiments are highly useful for ecological studies but can be affected by many simultaneous factors. This makes it difficult to infer plant responses associated with a single environmental factor. In contrast, plant growth chambers allow researchers to mechanistically determine what environmental conditions result in a specific plant response.

Growth chambers have been widely used in research (e.g. [6–9]); however it has been shown that although they are highly controlled, they are not uniform, which can lead to considerable degrees of variability in plant response data [10]. Variation in plant response data is normally present due to natural genotypic and phenotypic variation [9, 11, 12]; however this variation is compounded by what is termed ‘chamber effect’ i.e. variability in the data due to growing plants in different chambers. Long-term chamber experiments are probably more susceptible to ‘unwanted variation’ caused by chambers as environmental parameters can alter during experiments. Examples of this include light decay over time as light bulbs age, and changes in temperature, humidity and gas concentration as a result of sensor drift. Chamber effect is not only dependent on the duration of an experiment but also the type of experimental setup or design. These can be broadly divided into two types: within-chamber experiments and between-chamber experiments.

A within-chamber experiment involves all treatment conditions contained within a single plant growth chamber. For example, testing nutrient or water regimes across different individuals within a single chamber constitutes a within-chamber experiment and each individual plant/pot is a unit of replication. A chamber effect has been shown to be present with this experimental set up causing considerable variability in plant growth data [13–15]. This chamber effect is caused by spatial non-uniformity within a growth chamber and is dependent on the positioning of plants within the chamber. The chamber effect can substantially bias data results and the recommendations proposed to avoid this include increasing replication and randomising plant placement [13].

Between-chamber experiments involve one treatment condition per chamber and all plants within each individual chamber are grown under the same conditions (e.g. CO_2_ concentration, temperature or humidity treatments). Each chamber is considered one experimental unit and replication requires several chambers. Since all plants within a chamber are exposed to the same treatment, they are considered to be pseudo-replicates. However, similarly to within-chamber experiments, plants can still be subject to spatial variability, and therefore replicates and/or randomisation of plants are still required within each chamber. High variability in plant growth has also been shown for between-chamber experiments and recommendations to combat this involve increased replication, either by several chambers run in conjunction, or by time repeats [16]. Potvin and Tardif [16] demonstrated that plants grown in the same chamber but during different time periods exhibit the same chamber effect. As a result, they concluded that experiments should not be replicated in the same chamber twice. In contrast, Lee and Rawlings [10] suggest that there is a time chamber effect but also conclude that between-chamber experiments should be replicated over several chambers and/or over time.

Previous research has contributed to the knowledge of plant variability caused by chamber effects; however, this paper aims to address whether this variability is substantial enough to cause a significant difference in plant responses between chambers. If a chamber effect is strong enough to bias data, it could result in false interpretation and incorrect conclusions about a given treatment. Also, there are many types of plant growth chambers (shape, size, level of environmental control, airflow etc.) and different experimental set-ups; for this reason, making assumptions about appropriate experimental design for one’s own experiment based on another laboratory’s plant growth chambers can be misleading. In light of this, it is essential to establish if chamber effects exist in one’s own growth chambers by running a pilot study as outlined here prior to experimentation. This paper focuses on testing for ‘between-chamber effects’ by investigating which plant traits are most effective, timely and cost efficient to measure.

## Results and discussion

The purpose of the experiment was to investigate whether a chamber effect was present between eight Conviron (Winnipeg, Manitoba, Canada) BDW40 walk-in plant growth chambers and to determine which plant traits (if any) would be most effective for detecting it. Chamber effect may be the cause of minor variations between chambers so a relatively sensitive plant species must be used to detect such variations. For this reason, *Vicia faba* was chosen for its ability to respond to different environmental stimuli such as light [17, 18], atmospheric CO_2_ concentration and drought [19]. This species has also been shown to have increased stomatal sensitivity to [CO_2_] in chambers compared to those grown in greenhouses [20, 21]. To minimise variation between plants, *Vicia faba* plants were grown from seed in the same growing medium and pot size. Eighty seedlings were selected at random and placed in eight identical plant growth chambers, where light, temperature, humidity and atmospheric gases were controlled and monitored (Table 1).

**Table 1:** Plant growth chamber parameter settings

	Set point	CO_2_	Humidity	Temp day	Temp night	Light
		390 ppm	65 %	25 °C	15 °C	600 µmol
Chamber 1	Mean	391.60	64.97	24.68	15.01	597.88
	SD	14.22	1.01	1.10	0.10	7.95
Chamber 2	Mean	400.03	64.21	25.00	15.00	599.85
	SD	13.91	3.14	0.17	0.03	5.13
Chamber 3	Mean	401.76	64.96	23.93	15.12	598.02
	SD	11.96	2.22	0.83	0.55	10.17
Chamber 4	Mean	405.14	64.69	25.00	15.00	596.28
	SD	12.34	2.01	0.04	0.03	13.46
Chamber 5	Mean	400.09	64.74	24.64	15.01	598.52
	SD	14.48	1.86	1.22	0.09	7.11
Chamber 6	Mean	426.65	64.25	23.45	14.41	592.69
	SD	6.37	3.08	2.32	0.94	15.14
Chamber 7	Mean	392.10	62.29	24.82	15.01	599.30
	SD	10.46	5.36	0.92	0.11	5.59
Chamber 8	Mean	396.71	64.21	24.49	15.02	591.40
	SD	11.06	3.76	1.11	0.10	21.82

**Fig. 1:**
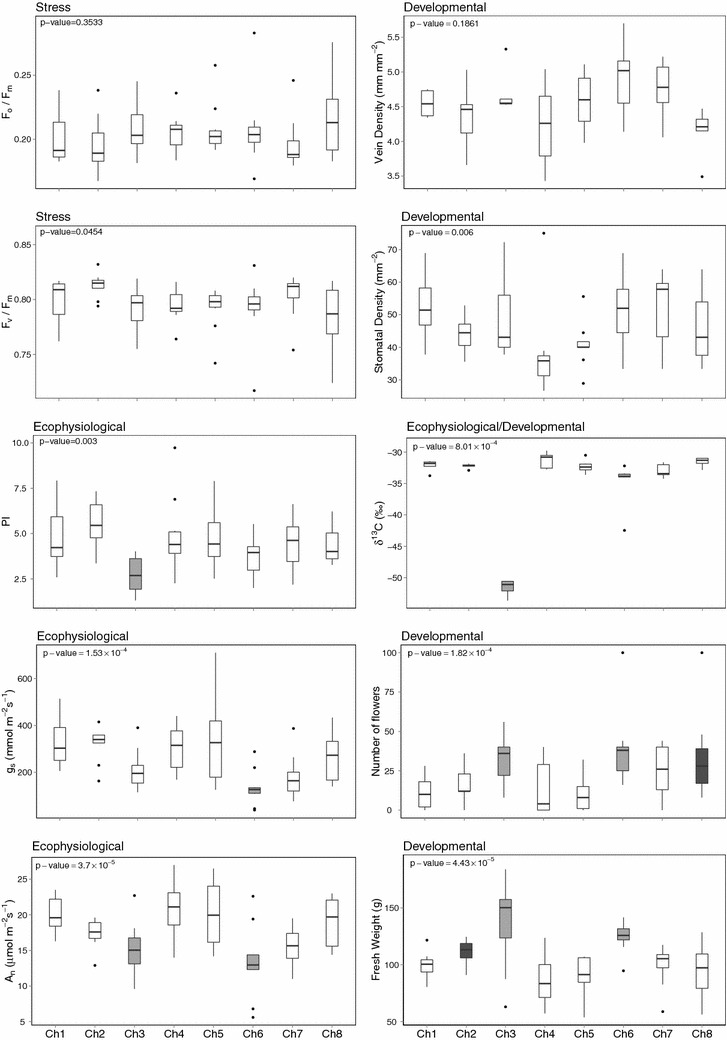
*Boxplots* (median, first [Q1] and third quartile [Q3], whiskers = 1.5 × IQR, *dots* outliers past whiskers) of *Vicia faba* L. plant traits. *Shaded boxes* display a significant difference after post hoc testing (FDR) with corresponding *p* values displayed. *Light grey* *resolvable* chamber effect, *dark grey* *unresolved* chamber effect

**Fig. 2:**
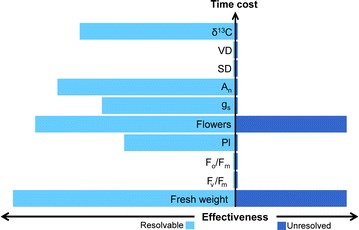
Measured traits of *Vicia faba* L. displayed in terms of their efficiency (ability to detect a chamber effect on *x* axis and time cost of analysis on *y* axis), where increased horizontal length of bars equals greater effectiveness and movement up the *y* axis equals increased time cost. *Light blue* *bars* *resolvable* chamber effect, *dark blue* *unresolved* chamber effect

**Fig. 3:**
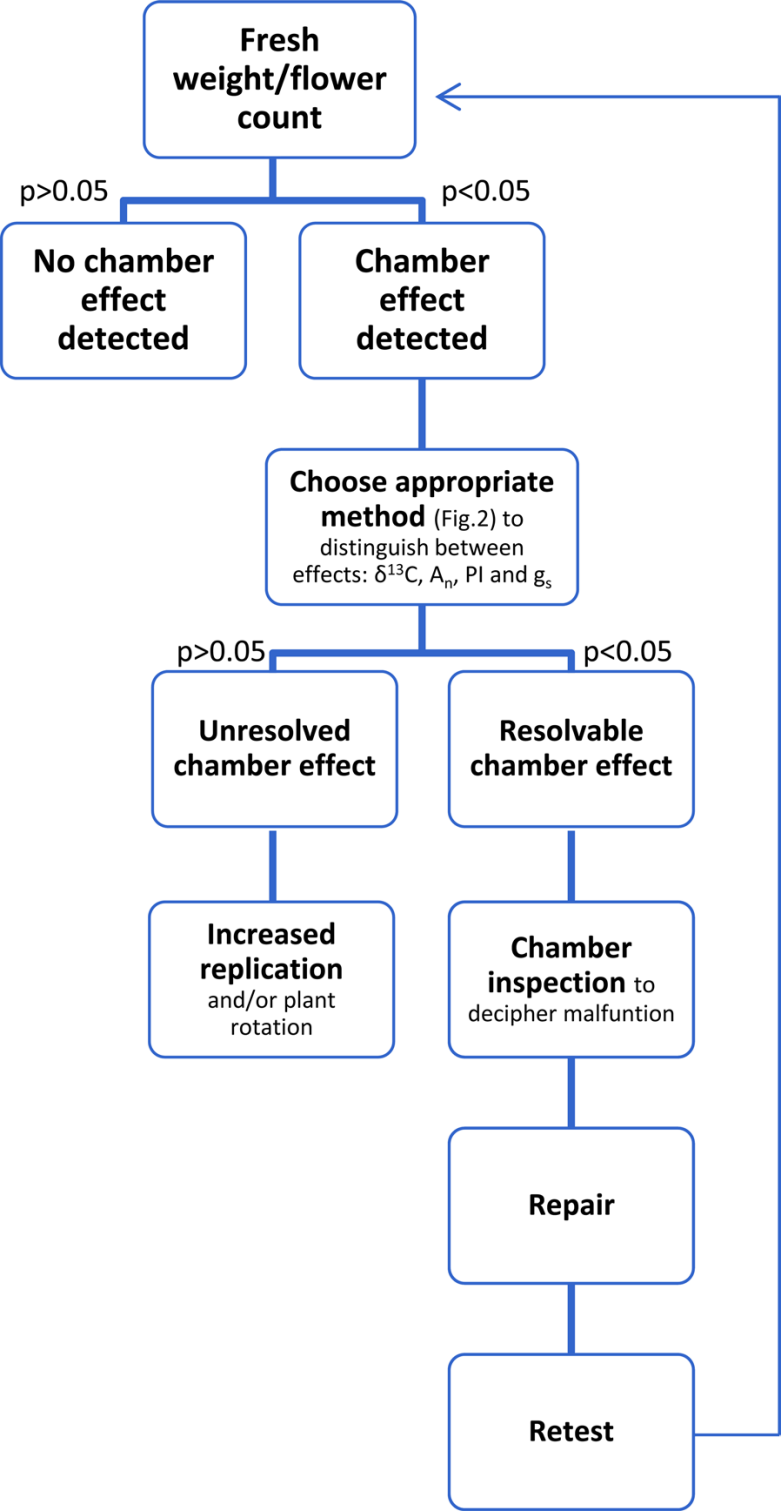
Stepwise method for detecting and distinguishing between *resolvable* and *unresolved* chamber effects in *Vicia faba* L. using fresh weight and flower count detection methods. *p* value refers to whether or not there is a significant difference (α = 0.05) between chambers after post hoc testing with FDR adjustments. Weight—above ground fresh biomass (g); *F*_*o*_ */F*_*m*_—minimum fluorescence in the absence of photosynthetic light/maximum fluorescence; *F*_v_/*F*_m_—variable fluorescence/maximum fluorescence; PI—total performance index; Flowers—number of individual flowers; g_s_—stomatal conductance (mmol m^−2^ s^−1^); A_n_—net rate of CO_2_ assimilation (µmol m^−2^ s^−1^); SD—number of stomata per mm^2^ leaf area; VD—vein length per unit area (mm mm^−2^); δ^13^C—ratio of leaf stable carbon isotopes ^13^C:^12^C (‰)

Four out of eight chambers (2, 3, 6 and 8) displayed a chamber effect (Fig. 1) in the form of statistically significant differences in the measured traits when a means comparison test was applied. The efficiency of each trait in detecting a chamber effect varied significantly and some traits were incapable of detecting any chamber effect (Fig. 2). For example, a chamber effect in both chambers 3 and 6 was detected by six separate measured traits (total performance index (PI), stomatal conductance (g_s_), net rate of CO_2_ assimilation (A_n_), stable carbon isotope composition (δ^13^C) of the leaves, flower count (number of individual flowers on inflorescences) and fresh weight) whereas a chamber effect in chambers 2 and 8 was detected by only one measured trait in each case (chamber effect in chamber 2 was detected by fresh weight, but by flower count in chamber 8) (Fig. 1). Although we found four separate chamber effects, two clear types can be identified: **‘***resolvable*’ chamber effects, defined as those caused by technical malfunctions in the chambers or chamber equipment that, once identified, can be repaired prior to commencement of experiments; or ‘*unresolved*’ chamber effects, which refer to effects of unknown source. Identifying a chamber effect as *resolvable* or *unresolved* can be challenging and typically demands observations from several plant traits (Fig. 3).

### Resolvable chamber effects

Fresh weight and flower count proved to be very effective in providing indications of a chamber effect. However, they are incapable of distinguishing between *resolvable* and *unresolved* chamber effects (Fig. 2); therefore, identification of *resolvable* chamber effects requires a combination of measured traits (Fig. 3). In this study, the *resolvable* chamber effect detected in chambers 3 and 6 demonstrates the potential troubleshooting capabilities of the different plant traits.

The stable carbon isotopes are especially useful because they allow the source carbon isotopes of CO_2_ to be tracked from the atmosphere to their final destination, which is plant tissues [22]. CO_2_ in the atmosphere is comprised of both ^13^C and ^12^C, with ^12^C being the more abundant isotope making up 98.9 % of total atmospheric CO_2_ [23]. The plant growth chamber source CO_2_ is supplied either from atmospheric CO_2_ or from CO_2_ gas cylinders, which may have a different carbon isotopic ratio; hence δ^13^C provides an ideal mechanism to test chamber effects caused by CO_2_ concentration and CO_2_ origin. The δ^13^C isotope data from this study revealed that *Vicia faba* individuals in six of the eight chambers showed no statistical difference in δ^13^C content; however, there was a difference in δ^13^C content in plants from chambers 3 and 6 (Fig. 1). In chamber 3, plant δ^13^C content was significantly lower (mean = −51.56, *p* value < 0.05) than in all other chambers (mean = −32.50). This large difference in chamber 3 leaf δ^13^C suggests that the isotopic ratio (^13^C:^12^C) of atmospheric CO_2_ in chamber 3 was lower compared to other chambers. An explanation for this could be that additional CO_2_ from gas canisters was injected into the chambers. When the experimental set point of CO_2_ is 390 ppm, ambient concentrations of CO_2_ enter the chambers via dampers (air vents). If a damper is inadvertently closed and/or if CO_2_ concentration in the chamber drops below set point level, CO_2_ from gas cylinders is injected into the chambers to maintain the set point. The CO_2_ used in compressed gas cylinders is produced from fertiliser and/or petrochemical processes (BOC, Industrial Gases, Ireland) and is highly depleted in ^13^C (Porter, unpublished data). If large amounts of CO_2_ were injected into chamber 3 from gas cylinders, this would lead to low δ^13^CO_2_ and result in very low leaf δ^13^C concentration. The low leaf δ^13^C thus indicated that source CO_2_ was likely to have originated from gas cylinders and this may have either raised the CO_2_ level much higher than 390 ppm (chambers are capable of reaching levels of 2000 ppm) or simply supplemented ambient CO_2_.

Combined consideration of all measured traits pointed towards a malfunction in the IRGA of chamber 3. The lack of any statistical differences in either *F*_*v*_*/F*_*m*_ (maximum photochemical efficiency of photosystem II) or *F*_*o*_*/F*_*m*_ (ratio of intrinsic fluorescence yield over maximum fluorescence yield under saturating light) indicated that the low A_n_ and PI values observed in chamber 3 plants did not result from photo-damage (Fig. 1). Nevertheless, these results could be interpreted in the context of a potential increased atmospheric CO_2_ concentration within chamber 3, possibly due to a faulty CO_2_ sensor. The lower photosynthesis observed could result from ‘high CO_2_′-induced photosynthetic downregulation often observed in plants grown at elevated [CO_2_]. Under these conditions plants tend to invest lower amounts of nitrogen into Rubisco [24] and show a reduction in both maximum carboxylation rates and electron transport rate supporting ribulose-1,5-bisphosphate (RuBP) regeneration [25]. Therefore the results from stable carbon isotopes, A_n_ and PI all point towards an increase in CO_2_ concentration in chamber 3, suggesting a malfunction of the WMA-4 infra-red gas analyser monitoring CO_2_ concentrations. Drifting of the zero set point is a common failure in gas analysers that could lead to injection of excess CO_2_ from the gas cylinders. Alternatively, a fault may have allowed the solenoid valve to open fully, injecting 2000 ppm CO_2_, yet this should have activated the chamber alarm which is set to ±20 ppm from the set point value.

Five separate plant traits detected a ‘chamber effect’ in chamber 6; these included δ^13^C, g_s_, A_n_, fresh weight and flower count (Fig. 1). Four of the five traits (excluding g_s_) also detected a chamber effect in chamber 3. Data trends were similar for both chambers, for example, greater number of flowers produced, higher fresh weight and decreased photosynthesis compared with the other chambers; thus it initially appeared that the origin of the chamber effect was similar for both chambers. However, despite the δ^13^C values from chamber 6 (mean value = −34.97, *p* value < 0.05) being significantly different to all other chambers, they were not found to be as low as chamber 3 (mean = −51.56) and fall closer in range to the remaining chambers (mean = −32.50). Thus, the small difference in δ^13^C values in chamber 6 leaves cannot be attributed to an influx of δ^13^C depleted CO_2_ from gas cylinders, and alternatively may reflect a plant response to a different type of chamber effect.

During the experiment, small white flakes were visible on the leaves in chamber 6. Upon completion of the study, the chamber was completely disassembled and all internal wall panels were removed. Corrosion of all metal components in the chamber had occurred due to mixing of SO_2_ gas with water from the overhead misting system in a previous study, which resulted in the formation of sulphuric acid and a build-up of sulphate salts. It appears that during the course of our experiment the salts escaped through the vents into the chamber and settled on the leaves. We suggest that, similarly to chamber 3, the chamber effect observed in this chamber was a *resolvable* one, resulting from severe corrosion and contamination of the chamber’s internal environment with sulphur dioxide gas.

### Unresolved chamber effects

Fresh weight and flower count identified a chamber effect in chambers 2 and 8 respectively (Fig. 1). This chamber effect seems weak as only a single trait was able to detect it in each case and the chambers in question were found to be statistically distinguishable from only 2–3 other chambers; chamber 2 differs only when compared with chambers 4, 5 and 6, and chamber 8 differs with only chambers 1 and 5. As there were no abnormalities detected for these chambers or chamber equipment upon inspection, we have concluded that chambers 2 and 8 have an *unresolved* chamber effect, i.e. an effect caused by unknown factors. The fact that this chamber effect is not mirrored in other plant traits and that both fresh weight and flower count could also detect an effect in chambers 3 and 6, suggests that these are very sensitive methods (Fig. 1).

### Recommendations for detecting *resolvable* and *unresolved* chamber effects

According to our results, measurements of fresh weight of above ground biomass and flower counts are the most effective, least expensive and quickest methods for detecting chamber effects (Fig. 2). However, neither of the two methods is able to distinguish between *resolvable* or *unresolved* chamber effects. Therefore, we propose that other measured traits should be used in conjunction with fresh weight and flower count (Fig. 2) to detect *resolvable* chamber effects; these include ratio of stable carbon isotopes (^13^C:^12^C) and/or net rate of CO_2_ assimilation (A_n_). For time efficiency, A_n_ is preferable as it is a relatively quick method (Fig. 2) but for experiments involving different atmospheric CO_2_ concentrations, stable carbon isotopes would be an appropriate choice because carbon isotope values give detailed information about CO_2_ origin and concentration. To detect *unresolved* chamber effects, only fresh weight and flower count are cost effective and time efficient methods.

A *resolvable* chamber effect, when detected, should be rectified prior to conducting experiments (Fig. 3). Where an *unresolved* chamber effect is detected, the solution requires increased experimental replication. This allows for good statistical analysis, both for existing chamber effects or effects that may arise during the course of an experiment. To avoid a potential within-chamber effect, plants should be randomly placed and rotated within chambers [13]. In order to avoid chamber effects for between-chamber experiments, plants can be rotated between replicate chambers during the course of an experiment [26–28]. By relocating the plants, each individual is subjected to multiple chambers, thus producing a smoothed data trend regardless of the presence of a chamber effect. Where possible it is preferable not to take this approach for two reasons: (1) the smoothed data values may not represent true values as all plants have now been exposed to any potential chamber effect through rotation; (2) although the smoothed trend minimizes chamber effect on individual plants, the range of variability in the data will most likely be significantly increased [29] as it may include the cumulative variation of each chamber, in the process losing information on which chamber is responsible for the chamber effect. In the absence of between-chamber plant rotation, chamber effects can be traced, and observed variability in data can be explained.

## Conclusions

Chamber effects exist for between-chamber experiments in the form of *resolvable* and *unresolved* effects. The former can be detected by many measured traits such as fresh weight, flower counts, gas exchange and stable carbon isotopes. In this experiment, *unresolved* chamber effects, although present, appeared to be weak and were only detected in *Vicia faba* by fresh weight measurements and flower counts. The underlying cause of *resolvable* chamber effects required investigation followed by repair of malfunctioning components and a subsequent pilot study conducted before any further experiments. To reduce the likelihood of a *resolvable* chamber effect occurring during the course of an experiment, we recommend that independent environmental sensors for CO_2_, O_2_, light and temperature be used on a regular basis to confirm that built-in chamber sensors have not drifted. As the cause of *unresolved* chamber effects is unknown, they cannot be easily rectified, but their induced variability can be minimised by between-chamber plant rotation and/or increased replication of experimental units.

## Methods

*Vicia faba* ‘Aquadulce Claudia’ seeds were sown individually into 0.5 L pots with Shamrock^®^ Multi-Purpose compost (Scotts Horticulture Ltd., Newbridge, Co. Kildare, Ireland). After 14 days germination, seedlings were transplanted to 1.5 L pots. Eighty randomly selected *Vicia faba* plants were grown in eight Conviron (Winnipeg, Manitoba, Canada) BDW-40 walk-in plant growth chambers in UCD Programme for Experimental Atmospheres and Climate (PÉAC) facility at Rosemount Environmental Research Station (i.e. ten plants per chamber). All chambers were fully cleaned to ensure equal transmission and reflection of light and all lightbulbs were replaced before initiation of the experiment. Two types of light bulbs were used: sixteen Venture metal halide (400 w, uniform pulse start high performance) lamps and sixteen Eveready E27 pearl incandescent (100 w rated at 1200 lumens) lamps. All chambers contained the same number and position of lightbulbs. The light spectrum in all chambers was measured using a light spectrometer (USV-650 Red Tide, Ocean Optics) to ensure that light quality was not a cause of chamber effect [30]. All chambers simulated the same conditions: 16/8 h photoperiod (06.00–10.00, light increased from 0 to 600 μmol m^−2^ s^−1^; 10.00–18.00, light at 600 μmol m^−2^ s^−1^; 18.00–22.00, light reduced from 600 to 0 μmol m^−2^ s^−1^); temperature 25 °C at midday and 15 °C at night; 390 ppm CO_2_; 65 % humidity (Table 1). Atmospheric CO_2_ concentration within the chambers was monitored using a PP-systems WMA-4 IRGA (PP-systems, Amesbury, Ma, USA). Each plant received 200 ml of water every 2 days for the first 3 weeks and 400 ml every 2 days thereafter. During the experiment, flower count was monitored daily (values represent total flower number during the growth period). Thirty days after initiation of the experiment gas exchange and chlorophyll fluorescence measurements were performed on the youngest fully expanded leaf of each plant. The experiment was conducted for 35 days, after which plant stems were severed from the roots at soil level and weighed (fresh weight). Fully expanded mature leaves were harvested for δ^13^C isotope, vein density and stomatal density analysis.

### Leaf clearing and staining for stomatal density and vein density

Leaves were processed following the protocol of Berlyn and Miksche [31]. Leaves were cleared using 5 % NaOH, rinsed three times with distilled water, then placed in 1 % bleach overnight. Leaves were rinsed three times again in distilled water and brought through an ethanol series (30, 50, 70, 100 %). They were then stained with Safranin O and Fast Green before being brought back through an ethanol series (100, 70, 50, 30 %) into distilled water and mounted onto glass slides using glycerol gelatine mounting medium.

### Stomatal density

Four cuticle images from each leaf (one leaf per plant, ten plants per chamber) were taken at 200× magnification using a Leica DM2500 microscope with Leica DFC300FX camera (Leica^®^ Microsystems, Wetzlar, Germany) and Syncroscopy Automontage (Syncroscopy, Cambridge, Cambridgeshire, UK) digital imaging software. A 0.09 mm^2^ square was superimposed onto each image using Syncroscopy AcQuis. Stomatal density was counted within this square using ImageJ software following a protocol from Poole and Kϋrschner [32]. The four counts per leaf were averaged and this value was used for statistical analysis.

### Vein density

Images from three leaf sections with an area of 1.25 mm^2^ each were taken at 50× magnification using a Leica DM2500 microscope with Leica DFC300FX camera (Leica^®^ Microsystems, Wetzlar, Germany) attached and Syncroscopy Automontage digital imaging software. Leaf minor vein density (quaternary and free-ending) was measured using ImageJ software from a total of 120 images (one leaf per plant, five plants per chamber).

### Stable carbon isotopes

One leaf from each plant and five plants per chamber were harvested, dried at 45 °C and ground to a fine uniform powder. Leaf samples were analysed for δ^13^C using a PDZ Europa ANCA-GSL elemental analyser interfaced to a PDZ Europa 20–20 isotope ratio mass spectrometer (Sercon Ltd., Cheshire, UK) at UC Davis Stable Isotope Facility, University of California, Davis, USA. Sample analysis included 10 % replication (one sample in ten was analysed twice to check for precision). The isotope δ values are expressed relative to international standards V-PDB (Vienna PeeDee Belemnite) where δ = (R sample − R standard/R standard) × 1000 and R = abundance ratio of the isotopes (i.e. ^13^C/^12^C). Instrumental error: ±0.03 ‰ (standard deviation).

### Gas exchange measurements

Net photosynthetic rate (A_n_) and stomatal conductance (g_s_) were recorded in situ, beginning 30 days after initiation of the chamber experiment. The measurements were performed using a CIRAS-2 gas analyser (PP-Systems, Amesbury, MA, USA) attached to a PLC6(U) cuvette fitted with a 1.7 cm^2^ measurement window, on the youngest, fully expanded leaf of each plant between 9:00 and 12:00 h. Even though CIRAS-2 allows the manipulation of light, humidity, CO_2_ and temperature, these environmental factors were not controlled; instead measurements were taken under chamber conditions in order to assess the in situ behaviour of the plants. For this purpose, the probe’s LED-head was removed so that the measurements were taken at growth chamber light intensity of ≈600 μmol m^−2^ s^−2^. Additionally, the CO_2_ concentration (390 μmol mol^−1^) and water vapour partial pressure (19.7 ± 1.3 mbar) used during the measurements were identical to those experienced by the plants in situ. Under these conditions average leaf temperature was 24.3 ± 0.7 °C and vapour pressure deficit was 0.85 ± 1.6 kPa. Upon clamping of the leaf in the cuvette, measurements were taken only after full stabilisation of A_n_ and g_s_, which typically took 3–5 min.

### Fluorescence measurements

Chlorophyll fluorescence measurements were performed on the youngest, fully expanded leaf of each plant, beginning 30 days after initiation of the chamber treatment. After dark-adapting the leaves for 1 h, a Pocket-PEA continuous excitation fluorimeter (Hansatech Instruments Ltd, Norfolk, UK) was used to measure their fast chlorophyll *a* fluorescence transients. Saturating light (≈3500 μmol m^−2^ s^−1^) was provided by a single high intensity red LED (peak at 627 nm) and chlorophyll fluorescence values were recorded from 10 μs to 1 s with data acquisition rates 10^5^, 10^4^, 10^3^, 10^2^ and 10^1^ readings in the time intervals of 10–300 μs, 0.3–3 ms, 3–30 ms, 30–300 ms and 0.3–1 s, respectively. The cardinal points of recorded polyphasic fluorescence kinetics [OJIP curves, cardinal points: fluorescence value at 20 μs (*F*_o_), fluorescence value at 300 μs ≤ (*F*_300μs_), fluorescence value at 2 ms (*F*_J_), fluorescence value at 30 ms (*F*_I_) and maximal fluorescence intensity (*F*_m_)] were then used to calculate the following parameters according to the JIP-test [33], as extended to include the effect of events related to the final electron acceptors of Photosystem I [34, 35]:

1. *F*_v_/*F*_m_ = (*F*_m_ − *F*_o_)/*F*_m_
2. *F*_o_/*F*_m_
3. Total Performance Index = PI_total_ = [V_j_ × *φ*_Po_/M_o_] × [*φ*_Po_/(1 − *φ*_Po_)] × [*ψ*_ET2o_/(1 − *ψ*_ET2o_)] × [*δ*_RE1o_/(1 − *δ*_RE1o_)]

where:

V_J_ = (*F*_J_ − *F*_o_)/(*F*_m_ − *F*_o_) is the relative variable fluorescence at 2 ms,

M_o_ = 4 × (*F*_300ms_ − *F*_o_)/(*F*_m_ − *F*_o_) is the initial slope of the OJIP curve,

*φ*_Po_ = 1 − *F*_o_/*F*_m_ is the quantum yield of primary photochemistry,

*ψ*_ET2o_ = 1 − V_J_ is the probability that a trapped electron will be transferred from Quinone A (Q_A_) to Quinone B (Q_B_),

*δ*_RE1o_ = (1 − V_I_)/(1 − V_J_) is the probability that an electron from QB will reduce the Photosystem I acceptors,

V_I_ = (*F*_I_ − *F*_o_)/(*F*_m_ − *F*_o_) is the relative variable fluorescence at 30 ms.

### Reproduction methods

Individual flower number was recorded weekly. Flowers consist of one standard, two wing and two keel petals; as each new flower emerged on an inflorescence, the standard petal was tagged to prevent the same flower being recorded twice over time. Total flower number per inflorescence and per plant was recorded for the duration of the experiment.

### Statistical analysis

Statistical analysis was performed in R (v.3.1.1). Where data was normally distributed, one-way ANOVA was performed. Kruskal–Wallis test for equal medians was performed for non-parametric data. Post hoc tests included: Tukeys pairwise multiple comparison test; Dunnett-Tukey–Kramer pairwise multiple comparison test; and Mann–Whitney pairwise test; each with a false discovery rate (FDR) adjustment to account for multiple comparisons.

## Abbreviations

*F*_*o*_*/F*_*m*_: Minimum, dark-adapted, intrinsic fluorescence yield (*F*_*o*_)/maximum fluorescence yield under saturating light (*F*_*m*_). *F*_*o*_*/F*_*m*_ is correlated with photo-damage in photosystem II
*F*_v_/*F*_m_: Variable fluorescence (*F*_*v*_ = *F*_m_ − *F*_o_)/maximum fluorescence yield under saturating light (*F*_m_). *F*_v_/*F*_m_ is a measure of maximum photochemical efficiency of photosystem II
PI: Total performance index: the product of the density of reaction centres, the quantum efficiency of primary photochemistry, the conversion of excitation energy in electron transport, and the quantum efficiency of reduction of photosystem I end acceptors [36, 37, 38]
g_s_: Stomatal conductance (mmol m^−2^ s^−1^)
A_n_: Net rate of CO_2_ assimilation (µmol m^−2^ s^−1^)
δ^13^C: Stable carbon isotope composition—the ratio of 13C/12C expressed relative to the PDB standard)
FDR: False discovery rate
